# Smyd3 Is Required for the Development of Cardiac and Skeletal Muscle in Zebrafish

**DOI:** 10.1371/journal.pone.0023491

**Published:** 2011-08-24

**Authors:** Tomoaki Fujii, Shin-ichiro Tsunesumi, Kiyoshi Yamaguchi, Sumiko Watanabe, Yoichi Furukawa

**Affiliations:** 1 Division of Clinical Genome Research, Advanced Clinical Research Center, Institute of Medical Science, The University of Tokyo, Japan; 2 Division of Molecular Developmental Biology, Institute of Medical Science, The University of Tokyo, Tokyo, Japan; Ludwig-Maximilians-Universität München, Germany

## Abstract

Modifications of histone tails are involved in the regulation of a wide range of biological processes including cell cycle, cell survival, cell division, and cell differentiation. Among the modifications, histone methylation plays a critical role in cardiac and skeletal muscle differentiation. In our earlier studies, we found that SMYD3 has methyltransferase activity to histone H3 lysine 4, and that its up-regulation is involved in the tumorigenesis of human colon, liver, and breast. To clarify the role of Smyd3 in development, we have studied its expression patterns in zebrafish embryos and the effect of its suppression on development using Smyd3-specific antisense morpholino-oligonucleotides. We here show that transcripts of *smyd3* were expressed in zebrafish embryos at all developmental stages examined and that knockdown of *smyd3* in embryos resulted in pericardial edema and defects in the trunk structure. In addition, these phenotypes were associated with abnormal expression of three heart-chamber markers including *cmlc2*, *amhc* and *vmhc*, and abnormal expression of myogenic regulatory factors including *myod* and *myog*. These data suggest that Smyd3 plays an important role in the development of heart and skeletal muscle.

## Introduction

The regulation of gene expression is achieved, in part, through epigenetic mechanisms that govern the association of transcription factors to DNA, and the nature of DNA packaging into chromatin [1]. The structure of chromatin containing nucleosome proteins and DNA is controlled dynamically through the modifications in histone tails, which include methylation, acetylation, phosphorylation and ubiquitination [2]. Among the modifications, methylation of H3K4, H3K36, and H3K79 is associated with transcriptional activation, while that of H3K9, H3K27, and H4K20 is associated with transcriptional repression. These methylations are catalyzed by histone methyltransferases containing a SET domain, and reversed by demetylases containing a jumonji domain. More than 60 SET domain-containing proteins have been identified so far, and among them, SET- and MYND-containing proteins termed SMYD proteins are evolutionally conserved from yeast to vertebrates. In human, there are five members of SMYD proteins; SMYD1, SMYD2, SMYD3, SMYD4, and SMYD5. Investigation on their catalytic activities disclosed that SMYD1, SMYD2 and SMYD3 have methyltransferase activities to histone H3 lysine4 [3]–[5], and that SMYD2 additionally exerts methylation on histone H3 lysine36 and p53 [6], [7].

We showed in our earlier reports that SMYD3 is up-regulated in colorectal, hepatocellular and breast cancer cells, and that its up-regulation plays a key role in the proliferation and survival of cancer cells. SMYD3 has a histone H3 lysine4 methyltransferase activity that is enhanced by HSP90α. Among adult tissues that we examined, SMYD3 is abundantly expressed in the testis and skeletal muscle [5]. Another report showed that it was ubiquitously expressed in zebrafish [8]. However, the physiological role of SMYD3 in development remains unknown.

Here, we investigated the expression of two forms of zebrafish *smyd3* transcripts during embryonic development and showed that Smyd3 plays a crucial role in the development of cardiac and skeletal muscle. These data may be useful for the understanding of diseases associated with cardiac abnormality or skeletal muscle defects.

## Results

### Identification of zebrafish *smyd3*

Using the BLAST program, we searched the zebrafish *smyd3* cDNA in the UCSC zebrafish database and obtained two sequences, ENSDART00000080847 and ENSDART00000105236, which shared 38% and 47% identity with human *SMYD3* cDNA, respectively. Except for a 144-nucleotide region being deleted from the middle of the sequence, the sequence for ENSDART00000080847 was identical to ENSDART00000105236, and both sequences were located on zebrafish chromosome 17. We termed the shorter ENSDART00000080847 transcript as *smyd3_tv1* and the longer ENSDART00000105236 transcript as *smyd3_tv2*. Comparison of these sequences with the zebrafish genome revealed that *smyd3* contains 12 exons, and that the two forms are generated by alternative splicing. The 144 nucleotides lacking in *smyd3_tv1* corresponds to a part of exon8 and the entire sequence of exon9. *Smyd3_tv1* encodes a deduced 380-amino acid protein, and *Smyd3_tv2* a deduced 428-amino acid protein containing an extra 48-amino acid insertion at position 252–299. The Smyd3_tv1 and Smyd3_tv2 proteins contain two conserved domains, a MYND domain (codons 49–87) and a SET domain (codons 156–239), and share 42% and 46% identity with human SMYD3 protein, respectively. However, a post-SET domain (codons 253–266) is included in Smyd3_tv2, but not in Smyd3_tv1 (Figure S1).

### Expression of *smyd3* in zebrafish development

**Figure 1:**
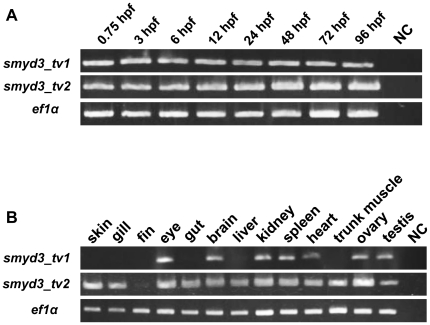
Expression of *smyd3_tv1* and *smyd3*_*tv2* in the developing zebrafish embryos and adult tissues (A) RT-PCR analysis was performed using *smyd3_tv1* and *smyd3_tv2-*specific primer sets, with RNA extracted from zebrafish embryos at 0.75, 3, 6, 12, 24, 48, 72, and 96 hpf. NC: negative control (RNase free water). Expression of *ef1α* served as an internal control. (B) Expression of *smyd3_tv1* and *smyd3_tv2* in various adult tissues.

To determine the expression of zebrafish *smyd3* in embryogenesis, we carried out RT-PCR using RNA extracted from embryos at different developmental stages and variant-specific primer sets. The analysis revealed that both forms of transcripts were expressed at all developmental stages from as early as 0.75 hpf to 96 hpf (Figure 1A). In adult zebrafish, RT-PCR detected *smyd3_tv1* transcripts in eye, brain, kidney, spleen, heart, ovary and testis, and *smyd3_tv2* in skin, gill, eye, gut, brain, liver, kidney, spleen, heart, muscle of the trunk, ovary and testis, but not in fin (Figure 1B), These data indicate that *smyd3_tv2* is specifically expressed in skin, gill, gut, liver, and muscle in the trunk.

### Knockdown of *smyd3* in developing embryos

**Figure 2:**
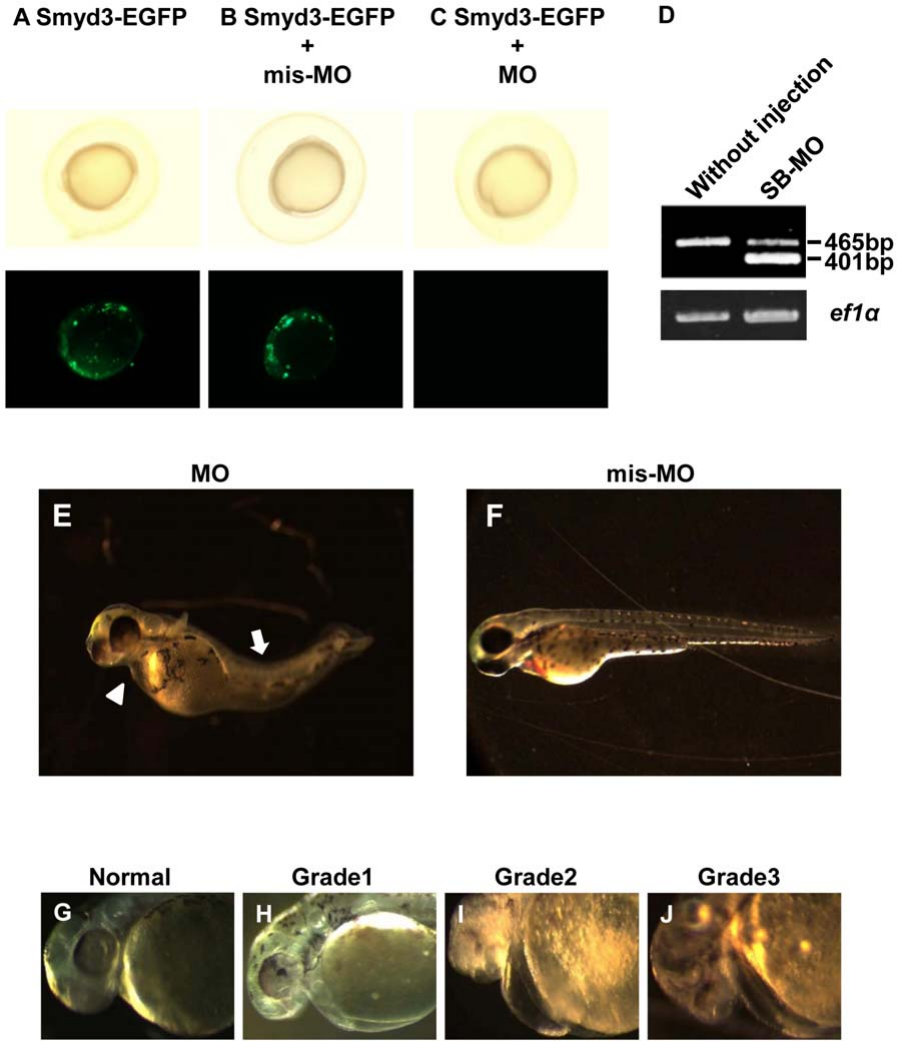
Effect of Smyd3 knockdown in zebrafish embryos by Smyd3-MO or Smyd3-SB-MO. (A, B, C, and D) Suppression of *smyd3* was examined at 10 hpf in embryos injected with *smyd3-EGFP* mRNA alone (A), *smyd3-EGFP* mRNA and Smyd3-mis-MO (B), and *smyd3-EGFP* mRNA and Smyd3-MO (C). Signals of EGFP were examined in the fluorescent macroscope (lower panel). The frequencies of EGFP-positive embryos were (A) 78.7%±2.3, (B) 83.7%±1.5, and (C) 17.7%±3.4. Data are shown as means±SEM. (D) Effect of Smyd3-SB-MO on *smyd3* transcripts. The wild-type transcripts were detected as a band at 465 bp and the aberrant transcripts at 401 bp. The lower panel shows the expression of *ef1α* as a control. (E and F) Phenotype of embryos injected with Smyd3-MO (E) or Smyd3-mis-MO (F) at 72 hpf. The pericardial edema (arrow head) and curved trunk (arrow) were observed in Smyd3 morphants (E). (G, H, I, and J) Morphological classification of heart defect. The degree of cardiac defect in the morphants was classified into three grades at 48 hpf. Grade1: Heart shows abnormality with mild looping defect and pericardial edema (H); Grade2: Heart shows abnormality with moderate looping and defect and pericardial edema (I); Grade3: Heart shows abnormality with string-like heart with severe pericardial edema (J). Normal: Normal heart (G). Embryos are shown in lateral view.

To determine the role of Smyd3 in the development of zebrafish embryos, we injected morpholino-oligonucleotides (MOs) designed to suppress Smyd3 (Smyd3-MO) or mutant MOs containing a five-nucleotide-mismatched sequence against Smyd3-MO sequence (Smyd3-mis-MO) into fertilized zebrafish eggs. We tested the effect of Smyd3-MO by co-injection with mRNA of *smyd3* fused with *EGFP* in zebrafish eggs. Expectedly, we observed significant decrease of EGFP signals by Smyd3-MO but not by Smyd3-mis-MO at 10 hpf (Figure 2A, B, and C). To confirm the knock-down effect of Smyd3, we additionally prepared MOs that block normal splicing (Smyd3-SB-MO) and performed RT-PCR using a *smyd3*-specific primer set that amplifies both normal and abnormal transcripts with exon-skipping. A band corresponding to normal splicing (465 bp) was detected in embryos injected with and without Smyd3-SB-MO, but a band corresponding to aberrant splicing (401 bp) was in embryos injected with Smyd3-SB-MO (Figure 2D). The abnormal transcripts of *smyd3_tv1 a*nd *smyd3_tv2* were deduced to result in the production of mutant proteins without its conserved region. These results suggested that Smyd3-MO and Smyd3-SB-MO effectively knocked down Smyd3. Interestingly, embryos injected with Smyd3-MO (termed Smyd3 morphants) exhibited pericardial edema and curved trunk (Figure 2E), which was not observed in embryos injected with Smyd3-mis-MO (Figure 2F). Of note, we could observe the normal morphology of the heart chambers (one atrium and one ventricle) and heartbeat in the morphants (Movie S1, Movie S2, and Movie S3).

**Figure 3:**
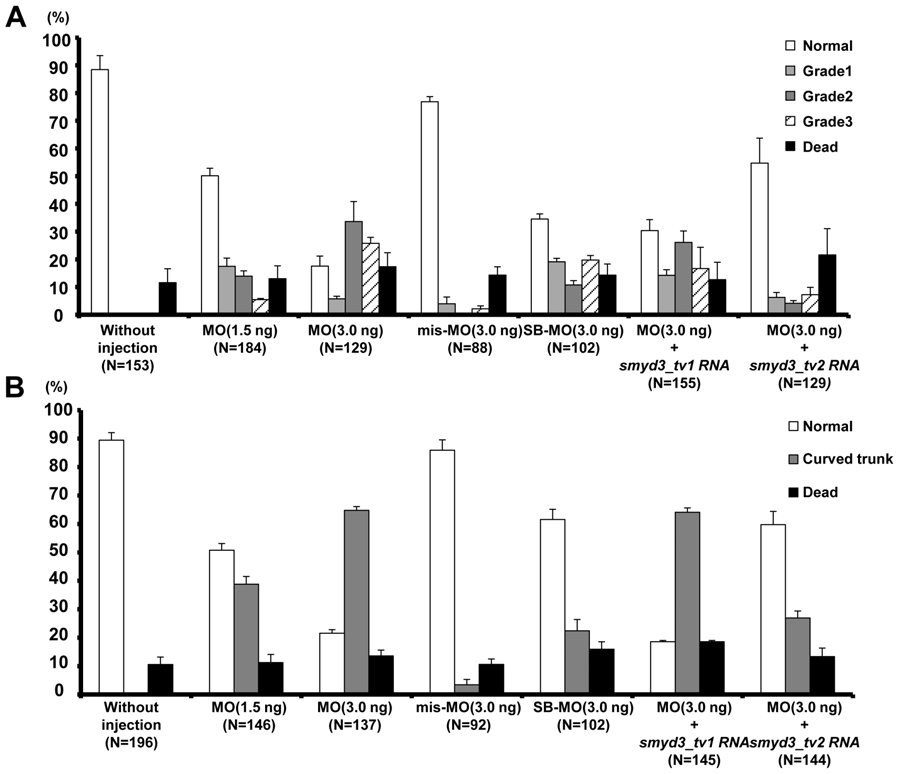
Frequencies of cardiac and trunk defects in Smyd3 morphants treated with different concentration of morpholinos, and with or without pre-injection with *smyd3_tv1* or *smyd3_tv2* mRNA. (A) Frequencies of heart defects in embryos injected with 1.5 or 3.0 ng of Smyd3-MO, 3.0 ng of Smyd3-mis-MO, 3.0 ng of Smyd3-SB-MO, 3.0 ng of Smyd3-MO in combination with *smyd3_tv1* or *smyd3_tv2* mRNA at 48 hpf. The histogram shows percentages of embryos with Normal (open box), Grade1 (light gray box), Grade2 (dark gray box), Grade3 (hatched box) or dead embryos (closed box). (B) Frequencies of curved trunk in embryos injected with 1.5 or 3.0 ng of Smyd3-MO, 3.0 ng of Smyd3-mis-MO, 3.0 ng of Smyd3-SB-MO, 3.0 ng of Smyd3-MO in combination with *smyd3_tv1* or *smyd3_tv2* mRNA at 48 hpf. The histogram shows percentages of embryos with Normal (open box), curved trunk (gray box), or dead embryos (closed box). Error bars represent the SEM. N is the total number of injected animals.

We classified the severity of heart defect into three grades at 48 hpf when cardiac looping was completed [9]; Grade1: a mild looping defect alone (Figure 2H); Grade2: a moderate looping defect with mild pericardial edema (Figure 2I); Grade3: a severe looping defect with pericardial edema (Figure 2J). Approximately 12% of without injection embryos died spontaneously, indicating the infertility of embryos in our culture condition. Injection with 3 ng of Smyd3-MO led to Grade2 and Grade3 defect in approximately 34% and 26% of embryos, respectively, while injection with 1.5 ng led to Grade2 and Grade3 defect in approximately 14% and 5% of embryos, respectively, suggesting a significant increase of cardiac defect (*p*<0.001) in a dose-dependent fashion (Figure 3A). On the other hand, Grade2 and Grade3 defects were found in 0% and 2% respectively, of embryos injected with Smyd3-mis-MO, indicating that Grade2 and Grade3 heart defects are significantly increased (*p*<0.001) in the Smyd3 morphants. Regarding trunk defect, injection with 1.5 ng and 3 ng of Smyd3-MO induced the curved trunk in approximately 40% and 65% of embryos, respectively, but only 3% of embryos developed the abnormality with 3 ng of Smyd3-mis-MO, which also showed a significant increase of curved trunk (*p*<0.001) in the morphants (Figure 3B). To confirm these phenotypes, we injected zebrafish eggs with Smyd3-SB-MO that suppressed normal splicing. As a result, the embryos injected with Smyd3-SB-MO consistently showed cardiac and muscle defects as observed in those injected with Smyd3-MO, although their severities and frequencies were less than Smyd3-MO (Figure 3A and B). These data suggested that Smyd3 plays a crucial role in the development of the heart and trunk.

To clarify the importance of Smyd3_tv1 and/or Smyd3_tv2 in cardiac and trunk defects of Smyd3 morphants, we performed a rescue experiment using *smyd3_tv1* and *_tv2* mRNA. Consequently, the cardiac defect and curved trunk in the Smyd3 morphants were significantly rescued by the injection with *smyd3_tv2* mRNA (*p*<0.001), but not by *smyd3_tv1* mRNA (Figure 3A and B). These data suggested that *smyd3_tv2* might play a major role in the development of the heart and trunk.

### Expression of cardiac markers in Smyd3 morphants

**Figure 4:**
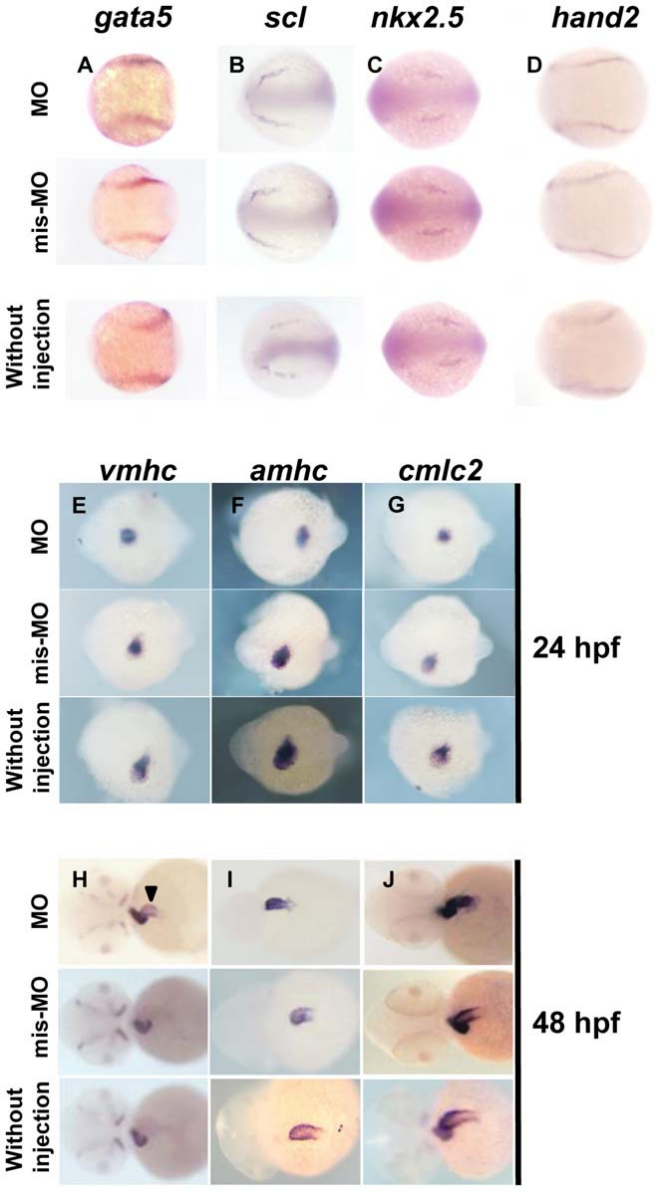
In situ hybridization analysis of ALPM and cardiac chamber markers. (A, B, C, and D) Expression *gata5*, *scl*, *nkx2.5* and *hand2* in Smyd3 morphants, control embryos injected with Smyd3-mis-MO and without injection at 12 hpf. (E, F, G, H, I, and J) Expression of *vmhc*, *amhc*, *and cmlc2* in the morphants, control embryos and without injection embryos at 24 hpf (E, F, and G) and 48 hpf (H, I, and J). Arrowhead indicates abnormal *vmhc* expression in the atrium (H). Embryos are shown in dorsal view, anterior toward the left (A, B, C, D, E, F, and G). Embryos are shown in frontal view, dorsal toward the left (H, I, and J).

To further disclose the mechanism(s) of heart defect in Smyd3 morphants, we studied the expression of seven markers; four anterior lateral plate mesoderm (ALPM) markers including GATA-binding protein 5 (*gata5*), stem cell leukemia protein (*scl*), NK2 transcription factor related 5 (*nkx2.5*), and heart and neural crest derivatives expressed transcript2 (*hand2*), and three cardiac chamber markers including ventricular myosin heavy chain (*vmhc)*, atrial myosin heavy chain (*amhc*) and cardiac myosin light chain2 (*cmlc2*). The *gata5*, *scl*, *nkx2.5* and *hand2* are markers specific to ALPM, rostral ALPM, caudal ALPM and medial ALPM, respectively [10]. The three markers, *vmhc*, *amhc* and *cmlc2* are specific to ventricle, atrium, and both chambers, respectively, and they are expressed in the heart tube of zebrafish embryos at 24 hpf [9]. In situ hybridization demonstrated that at 12 hpf, the expression of *gata5*, *scl*, *nkx2.5* and *hand2* in Smyd3 morphants was similar to that in the control embryos injected with Smyd3-mis-MO or without injection (Figure 4A, B, C, and D), suggesting that Smyd3 is not involved in the early myocardial specification. At 24 hpf, the expression of *amhc* and *cmlc2* was slightly shifted to the left side in the control embryos (Figure 4F and G), illustrating the normal elongation of the heart tube toward the left ventral side of the embryos [9]. On the other hand, their expression was localized at the midline of the Smyd3 morphants (Figure 4F and G). At 48 hpf, *vmhc* was slightly expressed in the atrium of the morphants in addition to its abundant expression in the ventricle, but it was confined to the ventricle in the control embryos (Figure 4H). This abnormal expression of *vmhc* was observed in 9 of 13 morphants, but not in any of 11 controls. Furthermore, expression of *amhc* and *cmlc2* was enhanced in the atrium of morphants compared with the controls (Figure 4I and J). These findings indicate that cardiac defect in Smyd3 morphants may result from impaired maturation and/or delayed development of cardiomyocytes.

### Expression of myogenic markers in Smyd3 morphants

**Figure 5:**
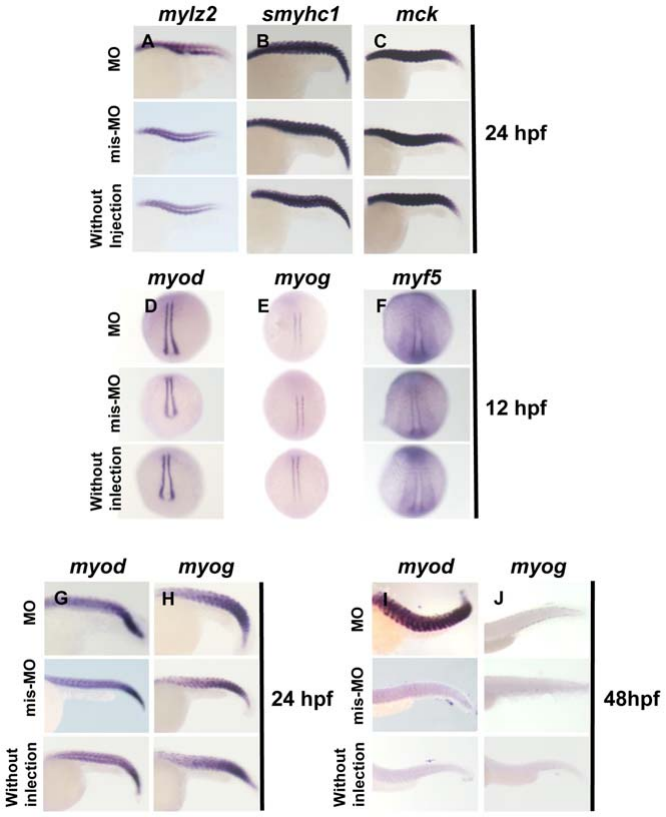
In situ hybridization analysis of terminal differentiation markers of skeletal muscle and myogenic regulatory factors. (A, B, and C) Expression of *mylz2*, *smyhc1* and *mck* in Smyd3 morphants, control embryos injected with Smyd3-mis-MO and without injection at 24 hpf. (D, E, F, G, H, I, and J) Expression of *myod*, *myog*, and *myf5* in Smyd3 morphants, control embryos injected with Smyd3-mis-MO and without injection at 12 hpf (D, E, and F), 24 hpf (G and H) and 48 hpf (I and J). Embryos are shown in lateral view, anterior toward the left (A, B, C, G, H, I, and J). Embryos are shown in dorsal view, anterior toward the top (D, E, and F).

To clarify the mechanism(s) underlying curved trunk, we investigated the expression of six markers; three terminal differentiation makers for skeletal muscle including skeletal muscle myosin light polypeptide 2 (*mylz2*), slow myosin heavy chain 1 (*smyhc1*), and muscle creatine kinase (*mck*), and three myogenic regulatory factors including myogenic differentiation (*myod*), myogenic factor 5 (*myf5*) and myogenin (*myog*). *mylz2*, *smyhc1*, and *mck* are differentiation markers for first muscle, slow muscle, and both slow and first muscle, respectively [11]. *myod* and *myf5* are expressed in the two lines of adaxial cells flanking the notocord of somites, while *myog* is expressed in the two lines of cells and paraxial mesoderm at 12 hpf. In situ hybridization clarified that the expression patterns of *mylz2*, *smyhc1*, *and mck* in Smyd3 morphants were indistinguishable from control embryos injected with Smyd3-mis-MO or without injection at 24 hpf when skeletal muscle differentiation is completed (Figure 5A, B and C). Expression of *myod*, *myog*, *and myf5* was not different between the morphants and controls at 12 hpf (Figure 5D, E, and F). The morphants and the controls maintained high levels of *myod* and *myog* expression in the trunks at 24 hpf (Figure 5G and H). Although the control embryos showed rapid decrease in *myod* and *myog* expression at 48 hpf, the morphants sustained significantly high *myod* and *myog* expression levels (Figure 5I and J). This sustained *myod* and *myog* expression was observed in all morphants depicting curved trunk. These data suggest that the abnormal trunk morphogenesis in Smyd3 morphants is not caused by the perturbation of muscle differentiation, but possibly by the deregulated expression of myogenic regulatory factors such as *myod* and *myog*.

## Discussion

Recent studies have unveiled that SMYD proteins are involved in the development of cardiac and skeletal muscle. For example, inactivation of *Smyd1*, also known as *Bop*, showed hypoplasia of the right ventricle in mice through disrupted maturation of ventricular cardiomyocytes [12], [13], and defect of muscle contraction in zebrafish through impaired myofibril organization [3]. SMYD1 expression is controlled by MYOD, Myogenin, and MEF2, transcription factors related to myogenesis, and is essential for *Hand2* expression that encodes a basic helix-loop-helix transcription factor expressed in cardiac muscle [12]. *Smyd2* was abundantly expressed in skeletal muscle and the face region during embryogenesis in *Xenopus laevis* [14]. Both *SMYD1* and *SMYD2* expression was gradually increased during porcine fetal muscle development [15]. In addition, muscle specific-depletion of *Drosophila Smyd4* led to the failure of eclosion resulting in late pupal death [16]. Besides SMYD proteins, other methyltransferases have been revealed to play a crucial role in muscle development. EZH2, a polycomb protein containing a SET domain, controls skeletal muscle differentiation through transcriptional repression of SRF and MYOD [17]. PEDM1 or Blimp-1/u-boot induces slow-twitch fiber-specific muscle differentiation by suppression of fast muscle-specific gene expression [18], [19]. The WDR5/ASH2L/MLL2 histone methyltransferase (HMT) complex activates MYOD, while SUV39H1 represses it [20], [21]. In addition to these reports, we have shown here that Smyd3 plays an important role in the development of cardiac and skeletal muscle.

We have additionally revealed that two forms of *smyd3* are expressed during zebrafish embryogenesis and in adult zebrafish. The two forms of transcripts encode proteins sharing most regions including the MYND and SET domains, but the short form (*smyd3_tv1*) lacks the post-SET domain. Since a post-SET was reported to enhance the methyltransferase activity coupled with another cystein in SET domain [22], the enzymatic activity of the long form (*smyd3_tv2*) may be higher than the short form. Consistent with this view, our rescue experiment showed that the long form (*smyd3_tv2*) seems to be more important than *smyd3_tv1* for cardiogenesis and trunk formation. We also found that their expression was different in several adult tissues; the expression of *smyd3_tv1* was almost diminished in the gill, skin, gut, liver and trunk muscle although *smyd3_tv2* was expressed ubiquitously in adult tissues. Therefore, the two forms of Smyd3 protein may have different roles in embryogenesis and adult tissues. Although the human ortholog SMYD3 protein contained a post-SET domain, a variant form termed SMYD3-NY lacking the N-terminal region was expressed in placenta, testis, ovary, kidney, spleen, and skeletal muscle [23].

In this study, we found that knockdown of zebrafish Smyd3 resulted in abnormal looping of heart tube accompanied by pericardial edema, which is similar to the Smyd1 morphants [3]. Heart development is governed by a complex gene regulatory network consisting of transcription factors, their co-factors, and downstream genes modulating cell fate specification, cell differentiation, cell proliferation, and cell migration. Among the network, transcription factors including Nkx2, GATA, Mef2, and Hand1/2 play a crucial role in early myocardial differentiation and morphogenesis [24], [25]. In situ hybridization demonstrated that Smyd3 morphants did not show abnormal expression of *gata5*, *scl*, *nkx2.5*, and *hand2*, at early stages but showed deregulated expression of *amhc*, *vmhc*, and *cmlc2*. These data may imply that Smyd3 is not involved in early specification of cardiomyocytes. It is of note that SMYD3 up-regulates the expression of *NKX2.5* in an embryonic kidney cell line HEK293 [5]. Unexpectedly, however, we found here that the expression of *nkx2.5* was unchanged in the Smyd3 morphants compared to control embryos. Since Smyd3 is a histone H3K4 methyltransferase, other H3K4 methyltransferase(s) such as Smyd1 may compensate the modification during heart development. Alternatively, *nkx2.5* may be regulated by different histone modification enzymes and/or transcription factors between kidney and cardiac muscle.

In addition to the heart defects, we have shown that Smyd3 morphants developed curved trunk, which was associated with sustained expression of *myod* and *myog* at a late developmental stage (48 hpf). Trunk skeletal muscle in vertebrates originates from a primary myotomal component of somites. Activation of myogenesis is regulated by a complex network comprising of the basic helix-loop-helix domain-containing myogenic regulatory factors (MRFs). Among the MRFs, Myod, the myogenic master transcription factor, is activated in adaxial cells adjacent to the notochord as early as 7–7.5 hours in zebrafish embryogenesis [26]. The *myod*-expressing cells expand in an anterior-to-posterior wave by 14.5 hpf, and markedly drop the *myod* expression by 24 hpf. The sustained expression of *myod* in Smyd3 morphants might result from deregulation of its upstream regulator such as Pax3 [27], or an undetermined mechanism of Myod regulation. Notably, *Smyd1/Bop* is transcriptionally regulated by MEF2C in the developing heart [13], and serum response factor and myogenin in myogenesis. From these data, it is tempting to speculate that Smyd3 is also regulated by MRFs and that inhibition of Smyd3 may activate Myod through a negative feedback loop. Since *myod* is known to enhance the expression of *myog*, the sustained expression of *myog* is likely due to the elevated expression of *myod*. Although additional studies are needed to clarify the mechanism(s) by which Smyd3 is implicated in muscle development, our findings should be a starting point for elucidating the roles of Smyd3 in myogenesis. Although Smyd3 morphants depicted cardiac defect and curved trunk, cardiac and skeletal myogenesis seem to be normally accomplished in early stages. Therefore, Smyd3 may not be involved in cell specification or differentiation, but involved in maturation or proliferation of differentiated myogenic cells.

In the present study, we have shown that *smyd3* plays a crucial role for cardiac and skeletal muscle development. These findings will be helpful for the understanding of molecular mechanisms underlying the development of heart and skeletal muscle.

## Materials and Methods

### Maintenance of zebrafish

Zebrafish (*Danio rerio*) were purchased from a local pet shop, and maintained under a 14-h day/10-h night cycle at 28.5°C. Fertilized eggs were obtained by mating adult fish from our outbred colonies soon after the light was turned on. Embryos were staged according to hours post-fertilization (hpf) and morphological criteria [28]. In our university, approval from the institutional committee for animal experiments is not necessary when using fish.

### Reverse transcription-polymerase chain reaction (RT-PCR) analysis

Total RNA was extracted from embryos or adult tissues using TRIzol solution (Life Technologies, Carlsbad, CA). cDNA was generated using 0.5 µg of total RNA with Surperscript II reverse transcriptase (Life Technologies) and oligo (dT)_15_ primers (Life Technologies). PCR reaction was performed using the cDNA as template. Primers used for the amplification were as follows: 5′-CGTGGCCCGATCATAAGAGG-3′ and 5′-ACAGCTCATCCCAGTGCTGG-3′ for *smyd3_tv1*, 5′-GGAGCAATACCACTTCCGGTGT-3′, and 5′-GCACTCGCTCAGTCTCCTCT-3′ for *smyd3_tv2*, 5′-TCACCCTGGGAGTGAAACAGC-3′ and 5′-ACTTGCAGGCGATGTGAGCAG-3′ for *ef1α*, 5′-CCGGAATTCTGAAATGATGGAGGCTGTG-3′ and 5′-CGTCGTGCAGAGATGCTTCA-3′ for the assessment of Smyd3-SB-MO.

### Microinjection of morpholino-oligonucleotides (MOs)

All antisense morpholino-oligonucleotides (MOs) were designated and supplied by Gene Tools LCC (Philomath, OR). The sequence of wild type MO (Smyd3-MO) was 5′-CCTCTCCATAATCACAGCCTCCATC-3′, and that of mismatch MO (Smyd3-mis-MO) containing five nucleotide-mismatches (indicated by lowercases) was 5′-CgTgTCCATAATgACAcCCTgCATC-3′. The sequence complementary to the initiation codon is underlined. Smyd3-SB-MO was 5′-ACTTTCACCCCTGTTAAGAATAAAT-3′, which was designed to block appropriate splicing of *smyd3* mRNA by binding at the splice junction between intron1 and exon2. MOs were diluted to 0.5 ng/nl or 1.0 ng/nl with 1× Danieau buffer and the same volume (approximately 3 nl) was injected into the yolk of 1- to 2-cell stage fertilized zebrafish eggs using microinjector (IM-300; Narishige, Tokyo, Japan) as described elsewhere [29]. The embryos were anesthetized on ice and observed under a macro zoom microscope (MVX10; Olympus, Tokyo, Japan). To confirm the knockdown of Smyd3, we utilized mRNA encoding Smyd3-EGFP fusion protein. A part of *smyd3* cDNA corresponding to the first 100 amino acids was amplified by RT-PCR using a set of primers, 5′-CCGCTCGAGTGAAATGATGGAGGCTGTG-3′ and 5′-CCGGAATTCGGTGGGGATCCTCGGCTGGA-3′, and the PCR products were cloned in an appropriate cloning site of pEGFP-N2 plasmid (Clontech, Heidelberg, Germany) to create the Smyd3-EGFP fragment. The fragment was subcloned into pCS2+ vector (pCS2-Smyd3-EGFP) to generate capped *smyd3-EGFP* mRNA. One ng of capped *smyd3-EGFP* mRNA was injected in zebrafish eggs with 1.5 ng of Smyd3-MO or Smyd3–mis-MO. Plasmids expressing *smyd3_tv2* were additionally prepared by RT-PCR using a set of primers, 5′-CCGCTCGAGTGAAATGATGGAGGCTGTG-3′ and 5′-CCGCTCGAGGACAGTGTTTTTATTTGAAATTGGG-3′, and subsequent cloning of the product into an appropriate site of pcDNA3.1 plasmids (pc-Smyd3_tv2). Plasmids containing *smyd3_tv1* (pc-Smyd3_tv1) were generated from pc-Smyd3_tv2 by the deletion of 144 nucleotides using the Quick Change Site-Directed Mutagenesis kit II (Agilent Technologies, Santa Clara, CA). The primers used for the amplification were, 5′-ACAGACGTTCCCAGCACTGGGATGAGCTGTTGAAG-3′ and 5′-ACAGCTCATCCCAGTGCTGGGAACGTCTGTCTTTA-3′. Rescue experiments were performed by a pre-injection with 300 pg of capped *smyd3_tv1* or *_tv2* mRNA and a subsequent injection with MOs as described earlier [30], [31]. The capped mRNA was synthesized using a m7G(5′)PPP(5′) G (Roche, Mannheim, Germany) and T7 or SP6 RNA polymerase (Roche) with pc-Smyd3_tv1, pc-Smyd3_tv2 or pCS2-Smyd3-EGFP. Fisher's exact test was employed for the analysis, and *p*<0.05 was considered statistically significant.

### Whole mount in situ hybridization

For in situ hybridization, the following genes were used as cRNA probes: *gata5*, *scl*, *hand2* [25], *cmlc2*, *vmhc* [32], *amhc* [33], *mck*, *mylz2*, *smyhc1* [16], *myod*, *myf5* and *myog* [26]. cDNAs were amplified by RT-PCR and the products were cloned into pcDNA3.1 plasmids (Life Technologies). Digoxigenin (DIG)-labeled RNA probes were transcribed using RNA DIG labeling mix (Roche) and T7 RNA polymerase (Roche). Whole mount in situ hybridization was carried out essentially as described elsewhere [29].

## Supporting Information

Figure S1 (A) Multiple alignment of human SMYD3, zebrafish *smyd3_tv1* and *tv2* protein sequences using CLUSTAL W. MYND, SET, and post-SET domain are indicated as a solid line above the sequence. Identical residues are indicated by asterisks, conserved substitutions by colons, and semi-conserved substitutions by periods.
(TIF)

Movie S1 **Heartbeats of a control embryo without injection at 48 hpf.**
(WMV)

Movie S2 **Heartbeats of a control embryo injected with Smyd3-mis-MO at 48 hpf.**
(WMV)

Movie S3 **Heartbeats of a Smyd3 morphant at 48 hpf.**
(WMV)
