# Supplementary figures and images for: Smyd3 Is Required for the Development of Cardiac and Skeletal Muscle in Zebrafish
Source: PLoS One. 2011 Aug 24;6(8):e23491. doi: 10.1371/journal.pone.0023491 (PMC3160858; doi:10.1371/journal.pone.0023491)

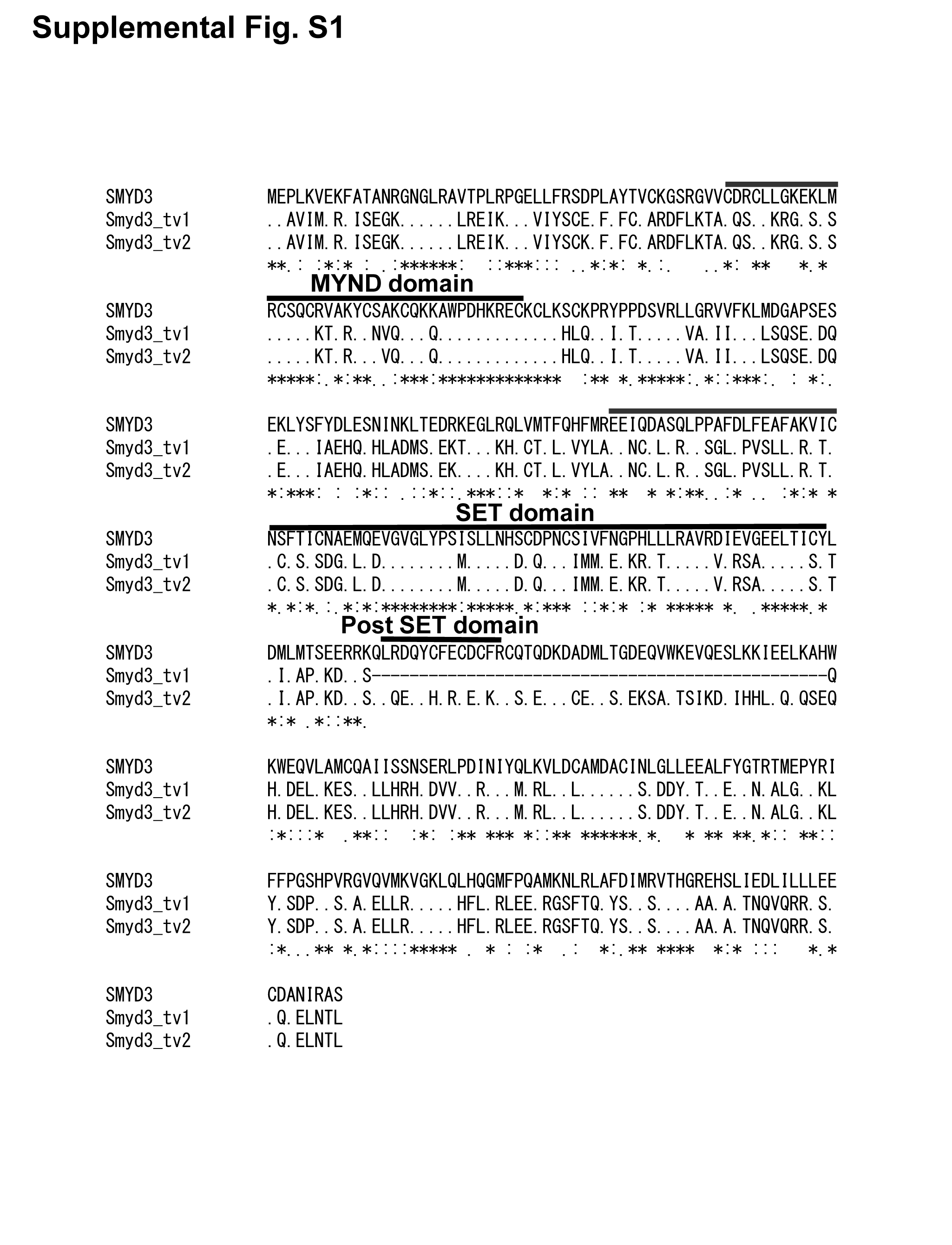

Supplement: Figure S1 — (A) Multiple alignment of human SMYD3, zebrafish smyd3_tv1 and tv2 protein sequences using CLUSTAL W. MYND, SET, and post-SET domain are indicated as a solid line above the sequence. Identical residues are indicated by asterisks, conserved substitutions by colons, and semi-conserved substitutions by periods. (TIF) [file pone.0023491.s001.tif]
